# Glypican-3 Enhances Reprogramming of Glucose Metabolism in Liver Cancer Cells

**DOI:** 10.1155/2019/2560650

**Published:** 2019-11-06

**Authors:** Gebing Yao, Jikai Yin, Qing Wang, Rui Dong, Jianguo Lu

**Affiliations:** Department of General Surgery, The Second Affiliated Hospital, The Air Force Military Medical University, Xi'an, Shaanxi 710038, China

## Abstract

Glypican-3(GPC3) is a transmembrane protein which has been found to be frequently overexpressed on the surfaces of liver cancer (LC) cells, which contributes to both the growth and metastasis of LC cells. Recently, the expression of GPC3 has been reported to be inversely associated with glucose metabolism activity in LC patients, suggesting that GPC3 may play a role in the regulation of glucose metabolism in LC. However, the role of GPC3 in glucose metabolism reprogramming, as well as in LC cell growth and metastasis, is unknown. Here, we found that GPC3 significantly contributed to the reprogramming of glucose metabolism in LC cells. On the one hand, GPC3 enhanced the glycolysis of LC cells through upregulation of the glycolytic genes of Glut1, HK2, and LDH-A. On the other hand, GPC3 repressed mitochondrial respiration through downregulation of peroxisome proliferator-activated receptor-gamma coactivator 1-alpha (PGC-1*α*), which has been well known as a crucial regulator in mitochondrial biogenesis. Mechanistic investigations revealed that HIF-1*α* was involved in both GPC3-regulated upregulation of glycolytic genes of HK2, PKM2, and Glut1 and downregulation of mitochondrial biogenesis regulator PGC-1*α* in LC cells. Additionally, GPC3-regulated reprogramming of glucose metabolism played a critical role in the growth and metastasis of LC cells. *Conclusion*. Our findings demonstrate that GPC3 is a critical regulator of glucose metabolism reprogramming in LC cells, which provides a strong line of evidence for GPC3 as an important therapeutic target to normalize glucose metabolic aberrations responsible for LC progression.

## 1. Introduction

Cancer cells preferentially use aerobic glycolysis to generate ATP and raw materials to meet their increased energetic and biosynthetic demands for cell growth and metastasis. This reliance on glucose under aerobic conditions is a hallmark of cancer cells, which has long been known as the phenomenon termed Warburg effect [1]. In liver cancer (LC) cells, enhanced glycolytic phenotype has also been observed [2–4]. Although intracellular signaling mediators such as PI3K/AKT, p53, and hypoxia-inducible factor (HIF) have been identified to play important roles in the reprogramming of aerobic glycolysis [5, 6], the molecular systems that are involved in the activation of these signaling pathways are still elusive, especially in LC. Given the crucial role played by increased aerobic glycolysis in tumor progression, it is necessary to identify core molecules or pathways that reprogram this process.

Glypican-3 (GPC3) is a member of the glypican family located at the extracellular side of the cell membrane, which has been identified as a potential therapeutic target in LC [7, 8]. No GPC3 expression can be detected in normal liver tissue, while GPC3 expression significantly increased in tumor tissues of LC. Currently, GPC3 has been used as a specific positron emission computed tomography (PET) imaging probe for LC detection [9, 10]. Besides, GPC3 has also been found to promote LC growth and metastasis [11, 12]. Overexpression of GPC3 in serum or tumor tissue predicts poor prognosis in patients with LC [13]. A recent study has indicated that GPC3 expression was associated with the glucose metabolism in LC patients [14], suggesting that GPC3 may play a role in the regulation of glucose metabolism in LC. However, the role of GPC3 in glucose metabolism reprogramming, as well as in LC cell growth and metastasis, is still unknown.

In the present study, we systematically investigated the role of GPC3 in the reprogramming of glucose metabolism in LC cells. Moreover, the effect of GPC3-regulated glucose metabolism in the growth and metastasis was also explored in LC cells.

## 2. Materials and Methods

### 2.1. Liver Cancer Cell Lines and Tissue Sample Collection

Human liver cancer (LC) cell lines of HLF, SNU-354, SNU-368, SNU-739, and HLE were obtained from the Cell Bank of the Chinese Academy of Sciences, Shanghai, China. Cells were maintained in RPMI-1640 medium or DMEM supplemented with 10% FBS (Hyclone). All LC cell lines were authenticated using short tandem repeat DNA testing by the FMMU Center for DNA Typing in 2015. In addition, 50 primary LC tissue samples were collected during surgical resection at the Second Affiliated Hospital of Fourth Military Medical University in Xi'an, China. All included patients were histologically confirmed as LC, and all of the samples were obtained with the patient's informed consent. The clinicopathologic features of the 50 LC patients are listed in Supplementary [Supplementary-material supplementary-material-1]. The study protocol was approved by the Ethics Committee of Second Affiliated Hospital of Fourth Military Medical University and performed in compliance with the Declaration of Helsinki.

### 2.2. Knockdown and Forced Expression of Target Genes

LC cells were transfected with expression vectors for GPC3 or siRNA against GPC3 using Lipofectamine 2000 following the manufacturer's instructions. GPC3 RNA interference sequences were 5′-CCUGUUUCCAGUCAUCUAUTT-3′ (si-GPC3#1) and 5′-CCUGAAAGUAUUUGGGAAUUUTT-3′ (si-GPC3#2). A scramble form was used as a control: 5′-UUCUCCGAACGUGUCACGUTT-3′. The pSilencer™ 3.1-H1 puro vector was used for the generation of shRNA expression vector targeting GPC3. A control shRNA was used as a silencing negative control. For overexpression of GPC3, the coding sequences of GPC3 were amplified from cDNA derived from HLF cells and cloned into the pcDNATM3.1vector.

### 2.3. Quantitative Real-Time PCR (qRT-PCR)

TRIzol reagent (Invitrogen) was used for the extraction of RNA from LC cell lines following the manufacturer's instructions. The quality of total RNA was assessed using an Agilent 2100 Bioanalyzer (Agilent Technologies, Palo Alto, CA). After that, a PrimeScript RT Reagent Kit (Invitrogen) was used for cDNA synthesis. PCR was carried out using a 2 × SYBR Green qPCR Master Mix (S-2014, US Everbright Inc). Primers used are listed in Supplementary [Supplementary-material supplementary-material-1]. The *β*-actin was used as a loading control, and the relative quantification of gene expression (fold change) was calculated using the 2^−ΔΔCT^ method.

### 2.4. Western Blotting

RIPA lysis buffer was used for the extraction of protein from LC cells following the manufacturer's instructions. A WonderOrange™ Protein Quantitation Kit (S-2014, US Everbright Inc) was used for protein concentration determination. Proteins were separated in SDS-PAGE gel and then transferred to polyvinylidene difluoride (PVDF) membranes. The membranes were blocked with 5% BSA and further incubated overnight at 4°C with specific primary antibodies. The membranes were washed with TPBS and incubated for 2 h at room temperature with secondary antibody and visualized with an enhanced chemiluminescence (ECL) kit (Millipore, Billerica, MA, USA). All primary antibodies used and their working concentrations are listed in Supplementary [Supplementary-material supplementary-material-1].

### 2.5. Immunohistochemistry

For immunohistochemistry (IHC) analysis, resected tumor tissues from 50 patients with LC were fixed in 10% formalin solution and embedded in paraffin. Slices of 4 *μ*m were prepared and then deparaffinized, rehydrated, and blocked for endogenous peroxidase with 0.3% H_2_O_2_. After antigen retrieval with hot citrate buffer (pH = 6) under pressure, the sections were blocked with 5% BSA and then probed with primary antibodies at 4°C overnight. Then, the sections were incubated with IHC kit (Invitrogen) and visualized using the peroxidase-conjugated streptavidin and diaminobenzidine, followed by counterstaining with hematoxylin. Finally, the IHC staining was scored by a pathologist blinded to all clinical data by assigning the percentages of positive cells and their staining intensities under a light microscope at a magnification of ×100. Briefly, the staining intensity was graded on a scale of 0–3 as follows: 0 is no staining, 1 is weak staining, 2 is moderate staining, and 3 is strong staining. The percentages of the stained area were scored as follows: 0 (0%), 1 (0%–25%), 2 (25%–50%), 3 (51%–75%), and 4 (75%–100%). The intensity points multiplied by the percentage points makes the final semiquantitative IHC score.

For prognosis analysis, the median IHC scores were chosen as the cutoff point for distinguishing the low and high GPC3 expression levels. For correlations between the expressions of GPC3 and metabolic enzymes, IHC scores <3 were considered as (−), between 3 and 6 were considered as (+), between 6 and 9 (6 is not included) were considered as (++), and >9 were considered as (+++).

All primary antibodies used and their working concentrations are listed in Supplementary [Supplementary-material supplementary-material-1].

### 2.6. Cell Proliferation and Colony Formation Assays

MTS cell viability assay was used for the determination of cell proliferation of LC cells with different GPC3 levels. Briefly, MTS reagent (Promega, G3581) was added to each well of 96-well plates. The absorbance was detected at 490 nm with an ELISA plate reader. The experiment was repeated independently three times.

For cell colony formation assay, 500 LC cells with different GPC3 levels were seeded to 6-well plate and cultured approximately 2 weeks until evident colony formation was observed. Then, the colonies in each well were visualized by staining with crystal violet. Finally, the total number of colonies was counted.

### 2.7. Cell Migration and Invasion Assays

LC cells with different GPC3 levels were seeded to six-well plates. When cells were grown to approximately 80–90% confluence, the cell layer was scratched with a plastic pipette tip in the middle of the well and then washed with serum-free culture medium to remove the detached cells. After culturing for 48 h, cells migrated into the wounded area were visualized and photographed.

For cell invasion assay, 200 *μ*l of LC cells with different GPC3 levels in serum-free medium were seeded into Matrigel-coated transwell chambers (BD Biosciences) at a density of 1 × 10^5^ cells/well, and 500 *μ*l culture medium containing 10% serum was added to the lower chamber. After incubation for 48 h, cells on the upper surface of the chamber were removed, and cells invade to the lower compartment were stained with 5% crystal violet. Finally, penetrated cells were photographed and counted in 5 random fields.

### 2.8. Glucose Uptake and Lactate Production Assays

LC cells with different GPC3 levels were seeded into 6-well plates at a density of 4 × 10^5^ cells/well. After incubation for 48 h, the culture medium was collected for glucose uptake with a glucose colorimetric assay kit (BioVision) following the manufacturer's protocol. The reduction of glucose concentration (glucose concentration at the beginning of cell culture minus the glucose concentration after 48 h of cell culture) in the culture media equates to the amount of glucose taken up by the liver cancer cells. For determination of the concentrations of lactate in the culture medium, a lactate assay kit (Sigma) was used and the detection was applied according to the manufacturer's protocol.

The final results of glucose uptake and lactate production were normalized on the basis of the total protein amounts of cells that were determined using a WonderOrange™ Protein Quantitation Kit (S-2014, US Everbright Inc).

### 2.9. In Vivo Tumor Growth and Metastasis Assays

A total of 1 × 10^7^ LC cells with different expression levels of GPC3 were subcutaneously implanted into the flank of four-week-old BALB/c nude mice (6 mice per group). Tumor volumes were measured weekly, mice were sacrificed at the fifth week, and tumors were harvested.

For in vivo metastasis assay, 5 × 10^5^ LC cells with different GPC3 levels were injected intravenously into the mice. The mice were euthanized, and the whole liver and lungs were harvested. The number of intraliver metastatic foci, which are macroscopically visible to the naked eye as white tumors, in hepatic lobes other than the injected lobes was counted. For the determination of the lung metastatic foci number, lungs were immediately fixed in neutral formalin 10%. After tissues were paraffinized, 5 *μ*m thick paraffin sections were prepared and stained with hematoxylin and eosin (H&E). The number of metastatic nodules was evaluated under light microscopy (Olympus Corporation, Tokyo, Japan) at 400x magnification.

All animal experimental procedures were conducted and approved by the Animal Care and Use Committee of the Second Affiliated Hospital of Fourth Military Medical University.

### 2.10. Statistical Analysis

Results are expressed as mean ± SEM. Unpaired *t*-test and one-way ANOVA followed by Fisher's least significant difference (LSD) test were used for comparisons between two groups or multiple comparisons with SPSS 17.0 software (SPSS, Chicago, IL), respectively. Correlations between measured variables were tested by Spearman rank correlation analysis. *P* < 0.05 was considered as statistically significant.

## 3. Results

### 3.1. GPC3 Enhanced the Warburg Effect in Liver Cancer Cells

**Figure 1:**
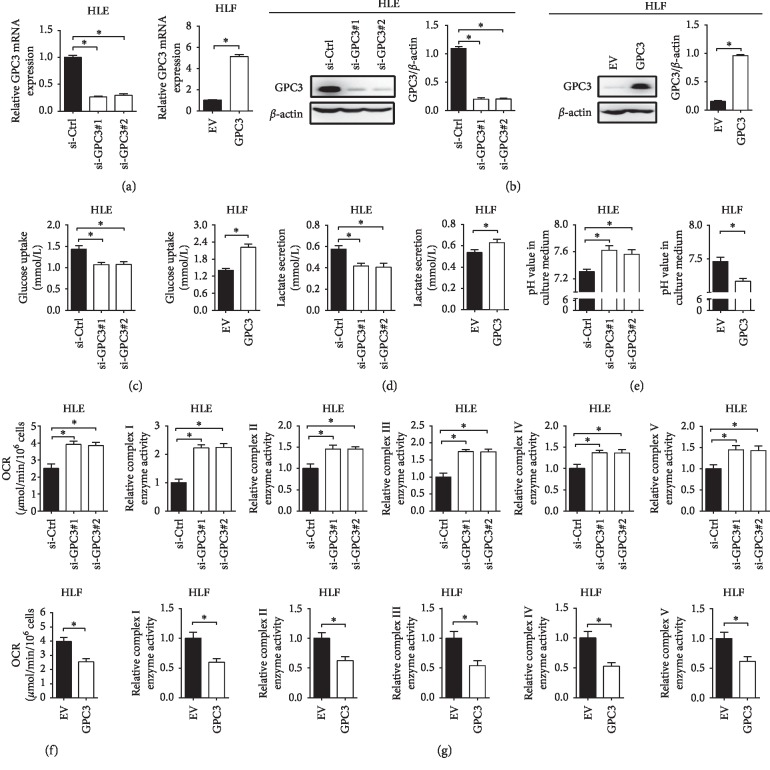
GPC3 enhanced the Warburg effect in LC cells. (a and b) Knockdown or overexpression of GPC3 in HLE and HLF cells was confirmed by quantitative real-time PCR (qRT-PCR) and western blot analysis at mRNA and protein levels. (c) Glucose uptake was measured in HLE and HLF cells transfected with siRNAs or expression vectors as indicated (si-GPC3#1 and si-GPC3#2, siRNAs against GPC3; siCtrl, control siRNA; GPC3, expression vector encoding GPC3; EV, empty vector). (d). Lactate production was measured in HLE and HLF cells with treatment as indicated. (e) The pH value in the culture medium was measured in HLE and HLF cells with treatment as indicated. (f) Oxygen consumption rate (OCR) was measured in HLE and HLF cells with treatment as indicated. (g) Relative enzyme activities of respiratory complexes I–V were measured in HLE and HLF cells with treatment as indicated. Data shown are the mean ± SEM from three independent experiments. ^*∗*^*p* < 0.05; ^*∗∗*^*p* < 0.01.

To study the role of GPC3 in the regulation of LC cell glucose metabolism, we established LC cell lines that differ only in their GPC3 status. HLE cells with relatively high GPC3 expression (Figures [Supplementary-material supplementary-material-1] and [Supplementary-material supplementary-material-1]) were transfected with nontargeting siRNA (siCtrl) or two siRNA targeting GPC3 (si-GPC3#1 and si-GPC3#2) for the establishment of GPC3 knockdown cell models, and HLF cells with relatively low GPC3 expression were transfected with an empty vector (EV) or an expression vector encoding GPC3 (GPC3) for the establishment of GPC3 overexpression cell models (Figures 1(a) and 1(b)). Our results showed that GPC3 knockdown HLE cells (si-GPC3#1 and si-GPC3#2) exhibited much lower cellular glucose uptake and lactate production, while higher pH value in the culture medium compared with the control cells (siCtrl). In contrast, HLF cells with GPC3 overexpression (GPC3) displayed significantly higher cellular glucose uptake and lactate production, while lower pH value in the culture medium compared with the control cells (EV) (Figures 1(c)–1(e)).

Increased glycolysis in tumor cells is always accompanied by decreased mitochondrial oxidative phosphorylation (OXPHOS) [15]. We thus hypothesized that mitochondrial OXPHOS in LC cells may be inhibited by GPC3. To test that, the effect of GPC3 on mitochondrial respiration was further examined. As shown in Figures 1(f) and 1(g), HLE cells with GPC3 knocked down (si-GPC3#1 and si-GPC3#2) exhibited a significantly higher oxygen consumption rate and increased activities of respiratory chain complexes I–V than control cells (siCtrl), whereas HLF cells with forced expression of GPC3 (GPC3) displayed a clearly lower oxygen consumption rate and decreased activities of respiratory chain complexes I–V than control cells (EV). Together, these results indicate that GPC3 plays an important role in the promotion of the Warburg effect in LC cells.

### 3.2. GPC3 Enhanced the Warburg Effect through Upregulation of Glycolytic Enzymes

**Figure 2:**
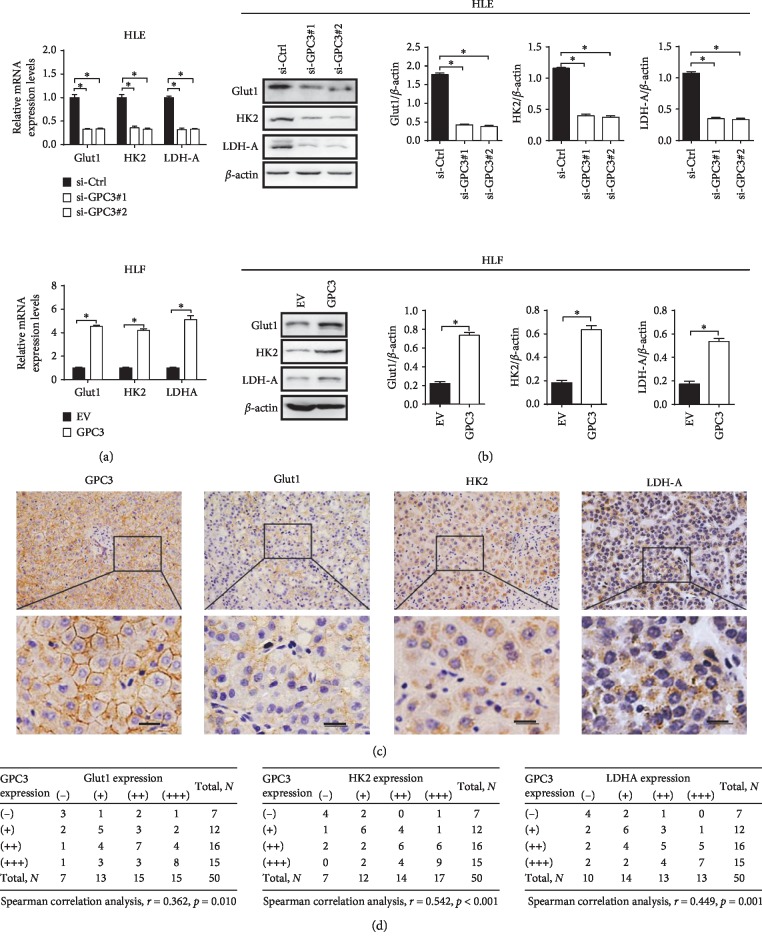
GPC3 enhanced the Warburg effect through upregulation of glycolytic enzymes. (a and b) qRT-PCR and western blot analyses for expression levels of glycolytic enzymes of Glut1, HK2, and LDH-A in HLE and HLF cells with treatment as indicated. (c) Representative immunohistochemical (IHC) staining images of GPC3, Glut1, HK2, and LDH-A in tumor tissues from 50 LC patients. (d) Spearman correlation analysis between expression levels of GPC3 and Glut1, HK2, and LDH-A in LC tissues. Data shown are the mean ± SEM from three independent experiments. ^*∗*^*p* < 0.05; ^*∗∗*^*p* < 0.01.

To explore the underlying mechanisms of GPC3 in the promotion of glycolysis, we first analyzed the expressions of the key glycolytic enzymes including Glut1, HK2, and LDH-A in HLE and HLF cells with different GPC3 levels. As shown in Figures 2(a) and 2(b), knockdown of GPC3 in HLE cells significantly downregulated the expression of HK2, PKM2, and Glut1, whereas overexpression of GPC3 in HLF cells clearly increased those glycolytic enzymes. To provide further support, the expressions of GPC3, Glut1, HK2, and LDH-A were determined in 50 LC tissue samples using immunohistochemistry (IHC) analysis (Figure 2(c)). Spearman rank correlation analysis indicated significant positive correlations between the expressions of GPC3 and the glycolytic enzymes of Glut1 (*r* = 0.362, *p*=0.010), HK2 (*r* = 0.542, *p* < 0.001), and LDHA (*r* = 0.449, *p*=0.001) (Figure 2(d)).

### 3.3. GPC3 Suppressed Mitochondrial OXPHOS through Downregulation of PGC-1*α*

**Figure 3:**
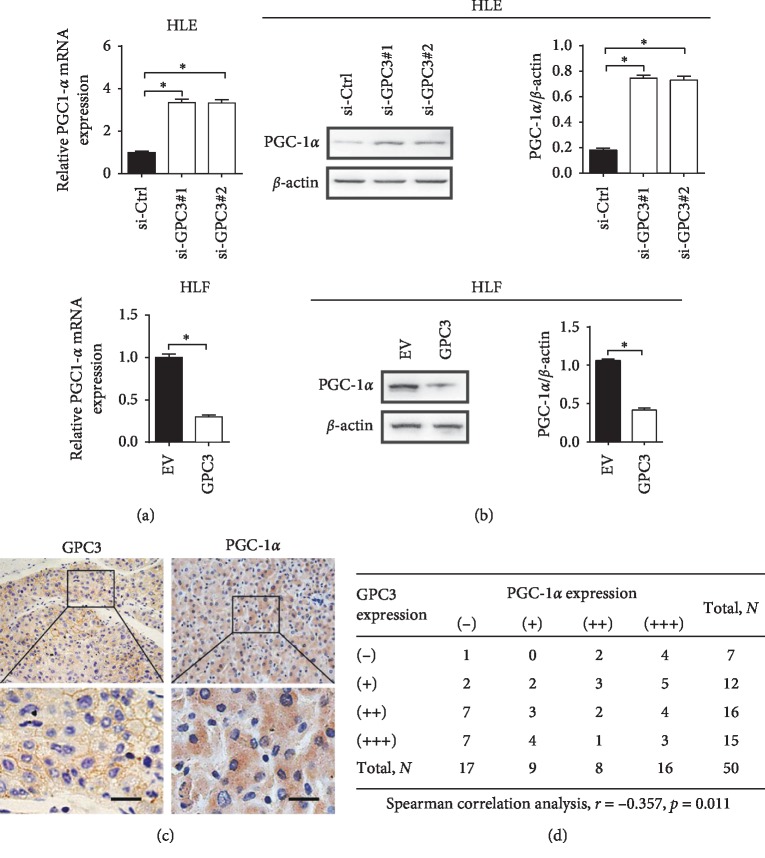
GPC3 suppressed mitochondrial OXPHOS through downregulation of PGC-1*α*. (a and b) qRT-PCR and western blot analyses for PGC-1*α* expression level in HLE and HLF cells with treatment as indicated. (c) Representative immunohistochemical (IHC) staining images of GPC3 and PGC-1*α* in tumor tissues from 50 LC patients. (d) Spearman correlation analysis between the expression levels of GPC3 and PGC-1*α* in 50 LC tissues based on the IHC staining results. Data shown are the mean ± SEM from three independent experiments. ^*∗*^*p* < 0.05; ^*∗∗*^*p* < 0.01.

Peroxisome proliferator-activated receptor-gamma coactivator (PGC-1*α*) has been characterized as a critical regulator of OXPHOS through facilitating mitochondrial biogenesis [16]. To determine whether PGC-1*α* is involved in the suppression of OXPHOS by GPC3, the expression of PGC-1*α* was firstly evaluated. As shown in Figures 3(a) and 3(b), the expression level of PGC-1*α* was significantly increased in HLE cells when GPC3 was knocked down, while clearly decreased in HLF cells when GPC3 was overexpressed. Spearman rank correlation analysis based on the IHC results of GPC3 and PGC-1*α* staining from 50 LC patients further indicated a significant negative correlation between the expressions of GPC3 and PGC-1*α* (*r* = −0.357, *p*=0.011) (Figures 3(c) and 3(d)).

### 3.4. HIF-1*α* Was Essential for the Effect of GPC3 in Glucose Metabolism Reprogramming

**Figure 4:**
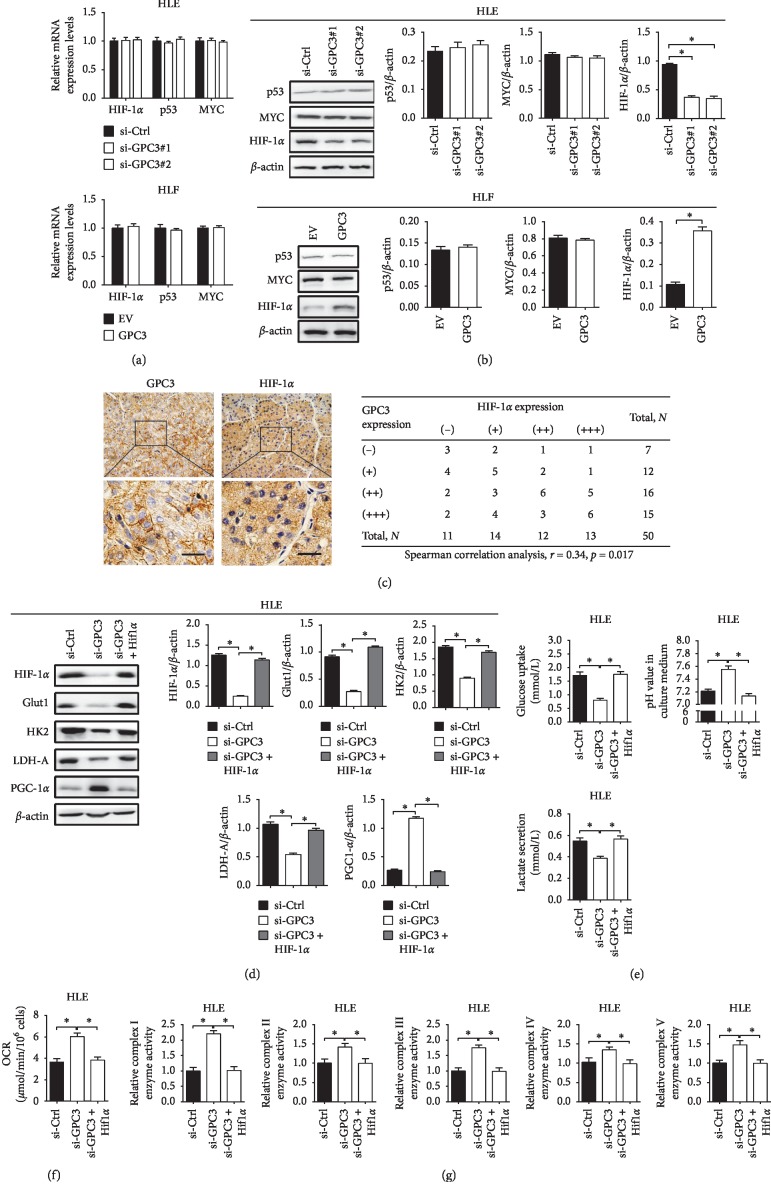
HIF-1*α* is essential for the effect of GPC3 in glucose metabolism reprogramming. (a and b) qRT-PCR and western blot analyses for expression levels of p53, MYC, and HIF-1*α* in HLE and HLF cells with treatment as indicated. (c) Spearman correlation analysis between the expression levels of GPC3 and HIF-1*α* in 50 LC tissues based on the results from IHC staining. (d) Western blot analysis for expression levels of HIF-1*α*, Glut1, HK2, LDH-A, and PGC-1*α* in HLE cells with treatment as indicated. (e) Glucose uptake, lactate production, and pH value in the culture medium were measured in HLE cells with treatment as indicated. (f) Oxygen consumption rate (OCR) was measured in HLE cells with treatment as indicated. (g) Relative enzyme activities of respiratory complexes I–V were measured in HLE cells with treatment as indicated. Data shown are the mean ± SEM from three independent experiments. ^*∗*^*p* < 0.05; ^*∗∗*^*p* < 0.01.

It has been well established that the transcriptional factors HIF-1*α*, p53, and c-MYC play important roles in the promotion of the Warburg effect in cancer cells [17]. Accordingly, the expressions of HIF-1*α*, p53, and c-MYC were first evaluated in LC cells with different levels of GPC3 by qRT-PCR and western blot analyses. As shown in Figure 4(a), no changes in HIF-1*α*, p53, and c-MYC at mRNA levels were observed when GPC3 was knocked down in HLE cells, while a significant decrease of HIF-1*α* at protein level (Figure 4(b)) was observed when GPC3 was knocked down in HLE cells. In contrast, forced expression of GPC3 exhibited an opposite effect in HLF cells. In addition, Spearman rank correlation analysis further indicated a positive correlation between the protein levels of GPC3 and HIF-1*α* (*r* = 0.34, *p*=0.017) (Figure 4(c)).

We then explored whether HIF-1*α* was involved in GPC3-regulated upregulation of glycolytic enzymes Glut1, HK2, and LDH-A and downregulation of OXPHOS factor PGC-1*α*. As shown in Figure 4(d), HIF-1*α* overexpression significantly restored the expressions of Glut1, HK2, and LDH-A suppressed by GPC3 knockdown, while decreased PGC-1*α* expression was upregulated by GPC3 knockdown. Moreover, GPC3 knockdown-mediated metabolic shift from glycolysis to OXPHOS was significantly reversed by overexpression of HIF-1*α* in HLE cells (Figures 4(e)–4(g)).

### 3.5. The Oncogenic Effect of GPC3 Is Dependent on Reprogrammed Glucose Metabolism in LC Cells

**Figure 5:**
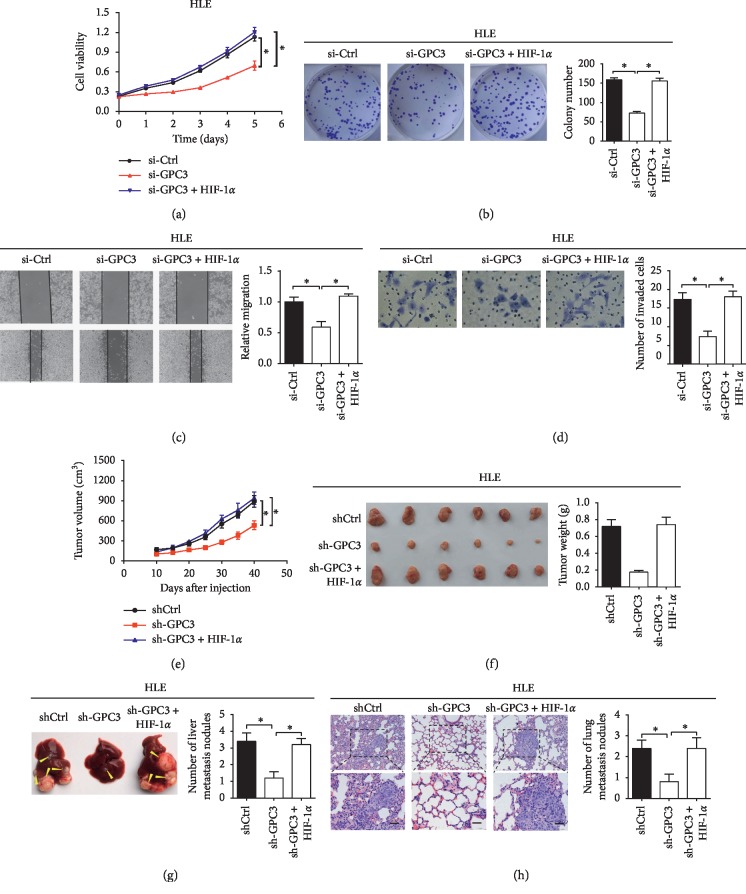
The oncogenic effect of GPC3 is dependent on reprogrammed glucose metabolism in LC cells. (a and b) MTS cell viability and colony formation assays in HLE cells treated as indicated. (c and d) Wound-healing migration and transwell invasion assays for migration and invasion abilities in HLE cells as indicated. (e) The subcutaneous tumor growth curve of HLE cells with different GPC3 expression levels in nude mice. (f) Dissected tumors from sacrificed mice and their weights are shown. (g and h) Incidence of intrahepatic and lung metastasis is shown. Arrow indicates metastatic foci. Data shown are the mean ± SEM from three independent experiments. ^*∗*^*p* < 0.05; ^*∗∗*^*p* < 0.01.

Considering the critical role of reprogrammed glucose metabolism in tumor progression [18], we hypothesized that GPC3-regulated glucose metabolism could be involved in the promotion of growth and metastasis in LC cells. To test that, HIF-1*α* was overexpressed in HLE cells with GPC3 knocked down. As shown in Figures 5(a)–5(d), forced expression of HIF-1*α* significantly reversed the suppressive effects of GPC3 knockdown on LC cell proliferation, colony formation, migration, and invasion in vitro. To further evaluate the potential role of GPC3-regulated glucose metabolism in LC growth and metastasis in vivo, we established stable cell lines by transfecting pcDNA3.1-HIF-1*α* plasmid into HLE with GPC3 expression stably knocked down to construct stable cell lines. GPC3 knockdown significantly suppressed the tumor growth (Figures 5(e) and 5(f)) and metastasis (Figures 5(g) and 5(h)) of LC cells, while forced expression of HIF-1*α* significantly reversed these effects. Taken together, GPC3 may exert its oncogenic effects by promoting glucose metabolism reprogramming in LC cells.

## 4. Discussion

Cancer cells exhibit high levels of glycolysis even under normal oxygen conditions to generate substrates for biomass generation and acidic microenvironment required for cell growth and metastasis [19, 20]. Understanding the molecular mechanisms underlying the metabolic switch from oxidative phosphorylation to glycolysis is critical for the development of new strategies for cancer therapy. In this study, we demonstrate that GPC3 significantly contributes to the reprogramming of glucose metabolism and thus the progression of LC mainly through HIF-1*α*-mediated upregulation of glycolytic enzymes Glut1, HK2, and LDHA and coordinately downregulation of the master regulator of mitochondrial biogenesis factor PGC-1*α*.

GPC3 has been found to be frequently overexpressed in LC cells, which is currently being used as a LC-specific positron emission computed tomography (PET) imaging probe for LC detection [8, 21]. In addition, GPC3 has been found to play important roles in the promotion of LC growth and metastasis [11, 12]. Recently, an association between the expression of GPC3 and glucose metabolism has been observed in the tumor tissues of LC patients, indicating that GPC3 may play a role in the regulation of glucose metabolism in LC cells [14]. Here, our results demonstrate that GPC3 acts as an important regulator of the Warburg effect by promoting glycolysis and inhibiting mitochondrial oxidative phosphorylation in LC cells.

Overexpressions of Glut1, HK2, and LDH-A have been found in many types of cancers, including LC [22–25]. Besides, increased aerobic glycolysis is commonly attributed to elevated expression of the key glycolytic enzymes [26]. Consistent with these results, we demonstrate that GPC3 promotes glycolysis of LC cells through upregulation of Glut1, HK2, and LDH-A. Moreover, we found that GPC3 can also inhibit PGC-1*α*-mediated mitochondrial oxidative phosphorylation, which provides new evidence for mitochondrial respiration inhibition not necessarily just as a consequence of increased glycolysis in tumor cells.

To date, several important intracellular signaling mediators, including p53, Myc, and HIF-1*α*, have been identified to play critical roles in the promotion of aerobic glycolysis through transcriptional activation of a wide range of genes involved in glycolysis [17]. However, it remains largely unclear what and how molecules on the cell surface are involved in the promotion of the Warburg effect. Our results showed that HIF-1*α*, but not p53 and Myc, was regulated by GPC3. It has been well established that the degradation by prolyl hydroxylases (PHDs) is one of the most common ways in the regulation of HIF-1*α* expression [27, 28]. In the present study, our data showed that knockdown of GPC3 resulted in decreased expression of HIF-1*α* at protein level but not at mRNA level, suggesting that GPC3 regulates the expression HIF-1*α* at the posttranscriptional level. In addition, our results indicated that the effect of GPC3 knockdown on the suppression of glycolytic phenotype was rescued by HIF-1*α* overexpression, implying that HIF-1*α* acts downstream of GPC3 to promote the glycolysis of LC cells.

Mounting research evidence has demonstrated that GPC3 plays important roles in the promotion of LC growth and metastasis [11, 12]. Considering that increased aerobic glycolysis generates substrates required for biomass generation and acidic microenvironment required for cell growth and metastasis, we propose that reprogrammed glucose metabolism may be involved in GPC3-regulated growth and metastasis of LC cells. As expected, our study demonstrated that overexpression of HIF-1*α* can robustly restore GPC3 silencing-mediated inhibition of growth and metastasis of LC cells.

In summary, our present findings demonstrate that GPC3 is a crucial regulator of glucose metabolism in LC cells, which provides a strong line of evidence for GPC3 as an important therapeutic target to normalize glucose metabolic aberrations responsible for LC progression.

## Data Availability

The data used to support the findings of this study are currently kept under wraps while the research findings are significant. Requests for data, 12 months after publication of this article, will be considered by the corresponding author on reasonable request.

## Supplementary Materials

Supplementary Materials Figure S1: the expression levels of GPC3 were determined by qRT-PCR (A) and western blot (B) analyses in HCC cell lines of HLF, SNU-354, SNU-368, SNU-739, and HLE. Table S1: clinicopathologic features of 50 liver cancer patients. Table S2: primers used in qRT-PCR analysis. Table S3: primary antibodies used in western blot and IHC analyses.

## References

[1] Cantor J. R., Sabatini D. M. (2012). Cancer cell metabolism: one hallmark, many faces. *Cancer Discovery*.

[2] Patterson A. D., Maurhofer O., Beyoglu D. (2011). Aberrant lipid metabolism in hepatocellular carcinoma revealed by plasma metabolomics and lipid profiling. *Cancer Research*.

[3] Videla L. A. (2009). Oxidative stress signaling underlying liver disease and hepatoprotective mechanisms. *World Journal of Hepatology*.

[4] Shang R.-Z., Qu S.-B., Wang D.-S. (2016). Reprogramming of glucose metabolism in hepatocellular carcinoma: progress and prospects. *World Journal of Gastroenterology*.

[5] Courtnay R., Ngo D. C., Malik N., Ververis K., Tortorella S. M., Karagiannis T. C. (2015). Cancer metabolism and the Warburg effect: the role of HIF-1 and PI3K. *Molecular Biology Reports*.

[6] Miller D. M., Thomas S. D., Islam A., Muench D., Sedoris K. (2012). c-Myc and cancer metabolism. *Clinical Cancer Research*.

[7] Montalbano M., Georgiadis J., Masterson A. L. (2017). Biology and function of glypican-3 as a candidate for early cancerous transformation of hepatocytes in hepatocellular carcinoma. *Oncology Reports*.

[8] Wang L., Yao M., Pan L.-H., Qian Q., Yao D.-F. (2015). Glypican-3 is a biomarker and a therapeutic target of hepatocellular carcinoma. *Hepatobiliary & Pancreatic Diseases International*.

[9] Zhou F., Shang W., Yu X., Tian J. (2018). Glypican-3: a promising biomarker for hepatocellular carcinoma diagnosis and treatment. *Medicinal Research Reviews*.

[10] Sham J. G., Kievit F. M., Grierson J. R. (2014). Glypican-3-targeted 89Zr PET imaging of hepatocellular carcinoma. *Journal of Nuclear Medicine*.

[11] Capurro M. I., Xiang Y.-Y., Lobe C., Filmus J. (2005). Glypican-3 promotes the growth of hepatocellular carcinoma by stimulating canonical Wnt signaling. *Cancer Research*.

[12] Wu Y., Liu H., Weng H. (2015). Glypican-3 promotes epithelial-mesenchymal transition of hepatocellular carcinoma cells through ERK signaling pathway. *International Journal of Oncology*.

[13] Zhou S., O’Gorman M. R., Yang F., Andresen K., Wang L. (2017). Glypican 3 as a serum marker for hepatoblastoma. *Scientific Reports*.

[14] Li Y.-C., Yang C.-S., Zhou W.-L. (2018). Low glucose metabolism in hepatocellular carcinoma with GPC3 expression. *World Journal of Gastroenterology*.

[15] Jose C., Bellance N., Rossignol R. (2011). Choosing between glycolysis and oxidative phosphorylation: a tumor’s dilemma?. *Biochimica et Biophysica Acta (BBA)—Bioenergetics*.

[16] Ventura-Clapier R., Garnier A., Veksler V. (2008). Transcriptional control of mitochondrial biogenesis: the central role of PGC-1*α*. *Cardiovascular Research*.

[17] Yeung S. J., Pan J., Lee M.-H. (2008). Roles of p53, MYC and HIF-1 in regulating glycolysis—the seventh hallmark of cancer. *Cellular and Molecular Life Sciences*.

[18] Li Z., Zhang H. (2016). Reprogramming of glucose, fatty acid and amino acid metabolism for cancer progression. *Cellular and Molecular Life Sciences*.

[19] Wu H. M., Li Y. M. (2017). In vitro antitumor activity of guttiferone-A in human breast cancer cells is mediated via apoptosis, mitochondrial mediated oxidative stress and reactive oxygen species production. *Journal of BUON*.

[20] Gatenby R. A., Gillies R. J. (2004). Why do cancers have high aerobic glycolysis?. *Nature Reviews Cancer*.

[21] Qin Z., Wang J., Wang Y. (2017). Identification of a glypican-3-binding peptide for in vivo non-invasive human hepatocellular carcinoma detection. *Macromolecular Bioscience*.

[22] Giatromanolaki A., Sivridis E., Arelaki S., Koukourakis M. I. (2017). Expression of enzymes related to glucose metabolism in non-small cell lung cancer and prognosis. *Experimental Lung Research*.

[23] Graziano F., Ruzzo A., Giacomini E. (2017). Glycolysis gene expression analysis and selective metabolic advantage in the clinical progression of colorectal cancer. *The Pharmacogenomics Journal*.

[24] Sanzey M., Abdul Rahim S. A., Oudin A. (2015). Comprehensive analysis of glycolytic enzymes as therapeutic targets in the treatment of glioblastoma. *PLoS One*.

[25] Lee N. C. W., Carella M. A., Papa S., Bubici C. (2018). High expression of glycolytic genes in cirrhosis correlates with the risk of developing liver cancer. *Frontiers in Cell and Developmental Biology*.

[26] Sydow G., Wildner G. P. (1971). Glycolytic enzymes and glycolysis in the lung and in primary lung neoplasms in man. *Acta Biologica et Medica Germanica*.

[27] Günter J., Ruiz-Serrano A., Pickel C., Wenger R. H., Scholz C. C. (2017). The functional interplay between the HIF pathway and the ubiquitin system—more than a one-way road. *Experimental Cell Research*.

[28] Masoud G. N., Li W. (2015). HIF-1*α* pathway: role, regulation and intervention for cancer therapy. *Acta Pharmaceutica Sinica B*.

