# Supplementary material for: Glypican-3 Enhances Reprogramming of Glucose Metabolism in Liver Cancer Cells
Source: Biomed Res Int. 2019 Nov 6;2019:2560650. doi: 10.1155/2019/2560650 (PMC6875211; doi:10.1155/2019/2560650)
Supplement: Supplementary Materials — Figure S1: the expression levels of GPC3 were determined by qRT-PCR (A) and western blot (B) analyses in HCC cell lines of HLF, SNU-354, SNU-368, SNU-739, and HLE. Table S1: clinicopathologic features of 50 liver cancer patients. Table S2: primers used in qRT-PCR analysis. Table S3: primary antibodies used in western blot and IHC analyses. [file 2560650.f1.docx]

**Supplemental information**

**Glypican-3 enhances reprogramming of glucose metabolism in Liver cancer cells**

**Supplementary Figures**

Figure S1. The expression levels of GPC3 were determined by qRT–PCR (A) and western blot (B) analysis in HCC cell lines of HLF, SNU-354, SNU-368, SNU-739 and HLE.

**
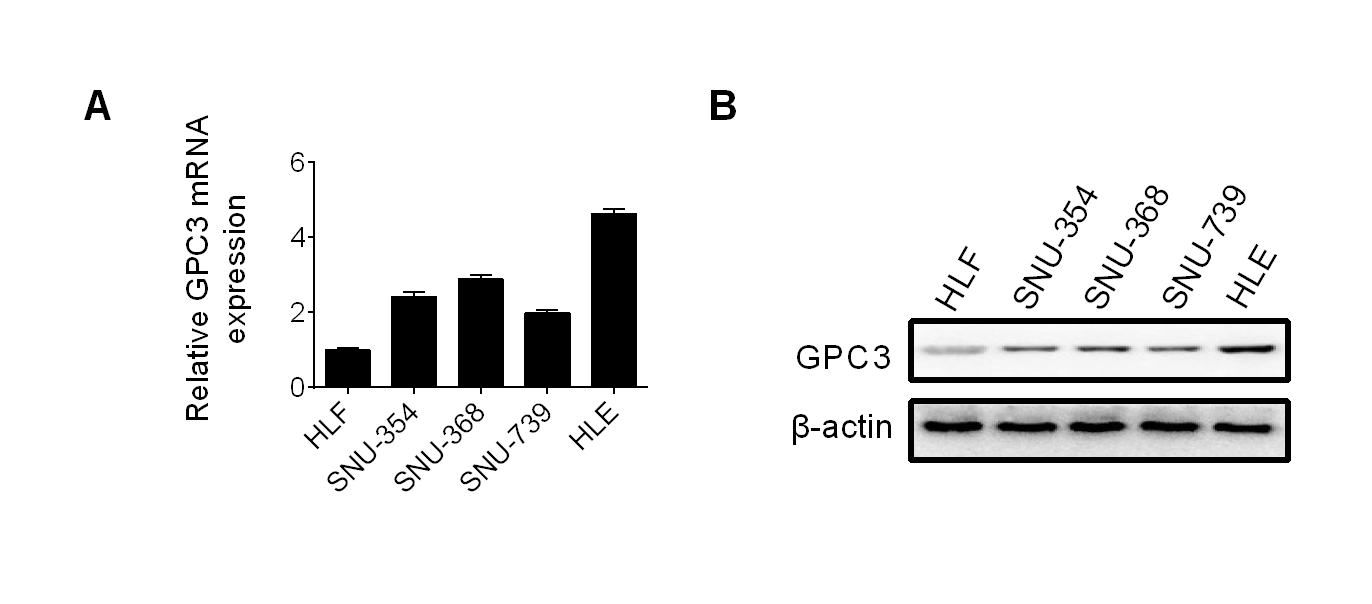
**

**Supplementary Tables**

**Supplementary Table 1. Clinicopathologic features of 50 liver cancer patients.**

| Variables | No. of cases (%) |
| --- | --- |
|  |  |
| All | 50 |
| Age |  |
| <55 | 22 (44%) |
| >=55 | 28 (56%) |
| Gender |  |
| Female | 14 (28%) |
| Male | 36 (72%) |
| HBsAg (hepatitis B surface antigen) |  |
| Negative | 9 (18%) |
| Positive | 41 (82%) |
| AFP (α -fetoprotein) (ug/ml) |  |
| <200 | 27 (54%) |
| >=200 | 23 (46%) |
| Maximum diameter of lesion |  |
| <5 | 41 (82%) |
| >=5 | 9 (18%) |
| PVTT (portal vein tumor thrombosis) |  |
| No | 45 (90%) |
| Yes | 5 (10%) |
| TNM (tumor-nodes-metastases) stage |  |
| I+ II | 39 (78%) |
| III+ IV | 11 (22%) |
| Differentiation grade |  |
| I+ II | 34 (68%) |
| III | 16 (32%) |
| Treatment |  |
| Hepatectomy | 37 (74%) |
| Hepatectomy + adjuvant TACE | 13 (26%) |

**Abbreviations**: TACE, transcatheter arterial chemoembolization

| **Supplementary Table 2. Primers used in qRT-PCR analysis** | | |
| --- | --- | --- |
| **Gene** | **Forward Primer** | **Reverse Primer** |
| *GPC3* | CCTTTGAAATTGTTGTTCGCCA | CCTGGGTTCATTAGCTGGGTA |
| *GLUT1* | GGCCAAGAGTGTGCTAAAGAA | ACAGCGTTGATGCCAGACAG |
| *HK2* | GAGCCACCACTCACCCTACT | CCAGGCATTCGGCAATGTG |
| *LDH-A* | ATGGCAACTCTAAAGGATCAGC | CCAACCCCAACAACTGTAATCT |
| *PGC1α* | TCTGAGTCTGTATGGAGTGACAT | CCAAGTCGTTCACATCTAGTTCA |
| *HIF1α* | GAACGTCGAAAAGAAAAGTCTCG | CCTTATCAAGATGCGAACTCACA |
| *p53* | CAGCACATGACGGAGGTTGT | TCATCCAAATACTCCACACGC |
| *MYC* | CTTCTCTCCGTCCTCGGATTCT | GAAGGTGATCCAGACTCTGACCTT |
| *β-actin* |  |  |

**Supplementary Table 3. Primary antibodies used in Western blot and IHC analysis.**

| **Antibody** | **Company (Cat. No.)** | **Working Concentration Dilutions** |
| --- | --- | --- |
| GPC3 | Proteintech (25175-1-AP) | WB: 1/800 IHC:1/100 |
| GLUT1 | Thermal (PA5-27246) | WB: 1/1000 IHC:1/200 |
| HK2 | Proteintech (22029-1-AP) | WB: 1/1000 IHC:1/200 |
| LDH-A | Proteintech (19987-1-AP) | WB: 1/1000 IHC:1/200 |
| PGC1α | Abcam (ab54481) | WB: 1/1000 IHC:1/200 |
| HIF1α | NOVUS (NB100-105) | WB: 1/500 IHC:1/100 |
| p53 | Proteintech (10442-1-AP) | WB: 1/800 |
| MYC | Proteintech (10828-1-AP) | WB: 1/1000 |
| β-actin | Beijing TDY(TDY051F) | WB: 1/5000 |
